# The MEK-ERK Pathway Is Necessary for Serine Phosphorylation of Mitochondrial STAT3 and Ras-Mediated Transformation

**DOI:** 10.1371/journal.pone.0083395

**Published:** 2013-11-29

**Authors:** Daniel J. Gough, Lisa Koetz, David E. Levy

**Affiliations:** New York University Langone School of Medicine, New York, NY, United States of America

## Abstract

Activating mutations in the RasGTPases are the most common oncogenic lesions in human cancer. Similarly, elevated STAT3 expression and/or phosphorylation are observed in the majority of human cancers. We recently found that activated Ras requires a mitochondrial rather than a nuclear activity of STAT3 to support cellular transformation. This mitochondrial activity of STAT3 was supported by phosphorylation on serine 727 (S727) in the carboxyl-terminus of STAT3. In this study we show that the H-Ras oncoprotein engages the MEK-ERK pathway to drive phosphorylation of STAT3 on S727, while phosphoinositide 3-kinase (PI3K) and mTOR activity were superfluous. Moreover, pharmacological inhibition of MEK reduced transformation by H-, K- or N-Ras. However, cells expressing a mitochondrially restricted STAT3 with a phospho-mimetic mutation at S727 were partially resistant to inhibition of the ERK pathway, exhibiting a partial rescue of anchorage-independent cell growth in the presence of MEK inhibitor. This study shows that the MEK-ERK pathway is required for activated Ras-induced phosphorylation of STAT3 on S727, that inhibition of STAT3 S727 phosphorylation contributes to the anti-oncogenic potential of MEK inhibitors, and that mitochondrial STAT3 is one of the critical substrates of the Ras-MEK-ERK- axis during cellular transformation.

## Introduction

Signal Transducer and Activator of Transcription (STAT)3 was originally described as a latent cytosolic transcription factor activated by phosphorylation on tyrosine (Y705) in response to stimulation by cytokines and growth factors (e.g., IL-6 family cytokines, IL-10, EGF, G-CSF, PDGF and HGF [1,2]). Phosphorylated STAT3 translocates to the nucleus and initiates the transcription of genes regulating cellular proliferation, angiogenesis, survival, metabolism and immune modulation [2], which, when persistently activated, are hallmarks of cancer. Constitutive over-expression or tyrosine phosphorylation of STAT3 is observed in many human cancers [3,4] and supports transformation in a range of cell culture and animal models. The best characterized activity of STAT3 in cancer is that of a constitutively tyrosine phosphorylated transcription factor as a result of de-regulated cytokine secretion (e.g IL-6 [5,6]) or mutations in tyrosine kinases including FLT3, EGFR, Src and JAK2 [3,7]. 

Phosphorylation of STAT3 on serine 727 (S727) also occurs in response to cytokine stimulation and enhances its transcriptional efficacy [8]. S727 phosphorylation of STAT3 has also been found to be constitutive in hematological malignancies (e.g., B-cell derived tumors and pediatric acute myeloid leukemia) [9,10]; however the only oncogenes that we are aware of that lead to S727 but not Y705 phosphorylation belong to the Ras family. The Ras oncogenes are the most common activating mutation in human cancer [11]. Mutation at codons 12, 13 or 61 locks Ras in a GTP bound active state, which initiates diverse signaling cascades including Raf-MEK-ERK, PI3K and Ral pathways that drive proliferation, survival and angiogenesis [11]. The Ras family of oncogenes (H-, N- and K-) do not directly stimulate STAT3 Y705 phosphorylation, yet STAT3 is still essential for transformation by these oncogenes [12]. This requirement for STAT3 in Ras-transformation was shown to require the S727 phosphorylated form of STAT3. 

The STAT3 serine phosphorylation site is embedded in a conserved PMSP sequence, which contains a mitogen activated protein kinase (MAPK) consensus target sequence (PXS/TP) [8]. However, multiple kinases are likely responsible for STAT3 S727 phosphorylation, depending on the nature of the activating signal [8]. The best characterized serine kinase responsible for STAT3 S727 phosphorylation is ERK (more specifically ERK2). ERK2 interacts with STAT3 [13], and treatment of cells with a MEK-ERK inhibitor (PD98059) blocks STAT3 S727 phosphorylation in response to IL-2 [14]. These data suggest that ERK is a candidate kinase to phosphorylated S727. There are also situations when ERK is not activated and yet S727 of STAT3 is phosphorylated, probably due to the activity of other serine kinases, including p38, JNK, PKCδ, SEK1, mTOR, or Rac1- or VAV-dependent kinases [8]. Ras oncogenes activate several serine kinases capable of phosphorylating STAT3 on S727; however the serine kinase that is required for STAT3 S727 phosphorylation downstream of the Ras oncogenes has not been definitively identified. 

STAT3 was recently shown to function in mitochondria, in addition to its canonical role as a nuclear transcription factor [12,15,16]. In mitochondria, STAT3 augments that activity of the electron transport chain through a mechanism that depends on S727 phosphorylation, and this function contributes to the ability of oncogenic Ras to transform cells. Mitochondrial STAT3 appears to contribute to the Warburg effect, a phenomenon in which transformed cells favor aerobic glycolysis over oxidative phosphorylation for the production of ATP [17].

In this study, we used genetic mutants and pharmacological inhibitors to show that H-, N- and K-Ras engage the Raf-MEK-ERK pathway to cause phosphorylation of STAT3 on S727, which is necessary for the anchorage-independent growth of transformed fibroblasts as colonies in soft agar. Moreover, we show that a STAT3 mutant that mimics S727 phosphorylation (S727D) is partially refractory to the block in transformation resulting from pharmacologic inhibition of the Raf-MEK-ERK MAPK pathway.

## Materials and Methods

### Antibodies and reagents

The following antibodies were obtained from commercial sources: STAT3 (4904), pY705 STAT3 (9145), pS727STAT3 (9134), pThr389 p70 S6 Kinase (9206), from Cell Signaling, Beverly, MA; Akt (SC-8312), pAkt (SC-7985), Tubulin, ERK1/2 (SC-94) and pERK1/2 (SC-7383), from Santa Cruz Biotechnology, Santa Cruz, CA, anti-Rabbit IRDye800 and anti-Mouse IRDye680 from LiCOR.

### Cloning and expression plasmids

The retroviral constructs (pBabe-puro) expressing H-RasV12 mutants H-RasV12/35S, and H-RasV12/40C were kind gifts from C. Der. N-RasV12 (pBabePuro-N-RasV12) and K-RasG12V (pBabePuro-K-RasV12) were the kind gift of A. Pellicer. Retroviruses (MSCV-GFP) expressing wild type STAT3 fused to an N-terminal mitochondrial targeting sequence from cytochrome c oxidase subunit VIII were constructed as previously described and generously provided by A. Larner [15]. The mitochondrially restricted STAT3 S727D mutant construct was generated by site directed mutagenesis using MTS-wild type STAT3 as a template using the following primer: gcaataccattgacctgcccatggacccccgcactttagatt. 

### Cell culture

3T3 immortalized STAT3-deficient mouse embryo fibroblasts were generated as described previously [12] and absence of STAT3 was confirmed by genomic PCR and western blotting. STAT3 mutants (MTS-STAT3 and MTS-STAT3 S727D) were stably introduced using retroviruses, as was stable expression of the oncogenes H-RasV12, N-RasV12 and K-RasG12. Transduced cell lines were maintained as pools of either drug-resistant cells or sorted GFP-positive cells, depending on the vector used, and the levels of expression of transduced STAT3 was determined by immunoblotting. Murine cell lines were grown in DMEM supplemented with 5% calf serum (CS) in a humidified incubator at 37°C and 5 % CO_2_. THP-1 cells were obtained from ATCC and grown in RPMI medium.

### Western blotting

Cells were lysed in a buffer containing 150mM NaCl, 50mM Tris (pH 7.4), 1mM EDTA, 0.5% Triton X-100, protease inhibitor cocktail (Sigma Aldrich), 1mM NaF, 1mM β-glycerophosphate, 1mM Na_3_VO_4_ and 1mM DTT. Protein preparations were resolved by 10% SDS-PAGE, transferred onto PVDF membranes (Millipore), and the membranes were incubated for 1 hour in 5% BSA in TBS-0.1% Tween-20 (20mM Tris-HCl pH 7.5, 500mM NaCl, 0.1% Tween-20). Blots were probed with primary antibodies diluted in TBS-0.1% Tween-20 buffer overnight at 4°C. The blots were washed and incubated with 1:15,000 dilution of fluor-conjugated secondary antibody (Li-Cor) and scanned using an Odyssey infrared scanner (Li-Cor).

### Soft agar assay

Cells (10^3^) were seeded in 0.3% agar, prepared in standard growth media. Cells were cultured in a humidified incubator at 37°C and 5% CO_2_ for 14 days and colonies greater than 0.2 mm^2^ were quantified. Each cell line was plated in three separate wells and each experiment was performed on three separate days. Data presented represent the mean of biological triplicates.

### Statistics

Significance of differences between data points means was calculated by the Student’s T test.

## Results

### The Raf-MEK-ERK pathway is required for S727 phosphorylation of STAT3 in Ras transformed cells

We have previously shown that STAT3 is essential for cell transformation by Ras oncogenes (H, N, and K-Ras), even in the absence of overt activation of the STAT3 pathway [12]. Indeed, the requirement for STAT3 is through a non-canonical pathway that does not involve STAT3 phosphorylation on tyrosine 705 that is characteristic of cytokine and growth factor signaling. In fact, tyrosine phosphorylation of STAT3 was not detected in Ras transformed cells. In contrast, we observed robust S727 phosphorylation, which has been shown to be critical for altered metabolic functions, including lactate production and the activity of the mitochondrial electron transport chain [12,15,18,19]. 

Ras engages numerous signaling pathways to elicit its tumorigenic activities, primarily the MEK-ERK, phosphoinositol-3-kinase (PI3K), and Ral pathways [20]. Activation of each single pathway is sufficient to induce limited cell transformation *in vitro*, and we have previously found that transformation of murine embryo fibroblasts (MEF) *in vitro* by each Ras-activated pathway is STAT3 dependent [12]. Moreover, while activation of each of these pathways in isolation is insufficient to fully transform cells, combined activation of the MEK-ERK and PI3K pathways transformed cells equivalently to H-RasV12 [12]. Because Ras transformation required STAT3 S727 phosphorylation, it was of interest to determine which Ras signaling pathway was responsible for this post-translational modification.

**Figure 1:**
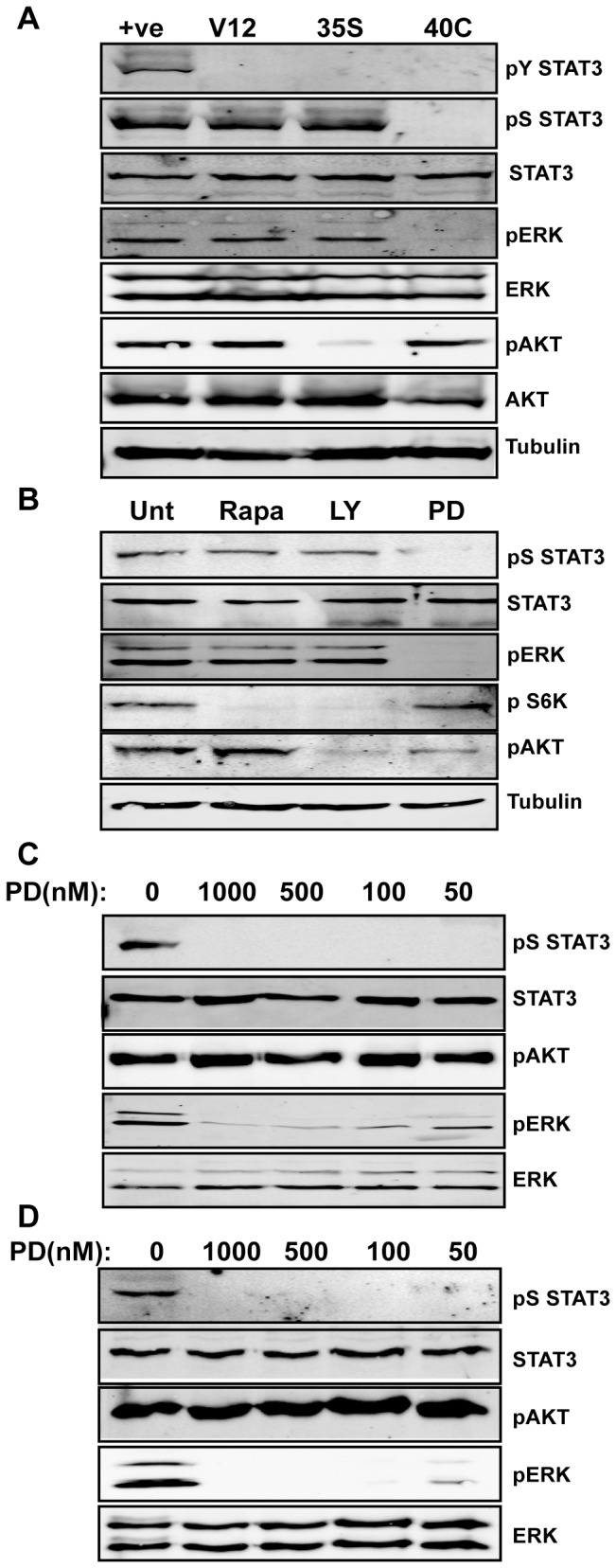
H-Ras phosphorylates STAT3 on S727 via the MEK-ERK pathway. (**A**) Protein extracts from MEFs transformed with H-RasV12 (V12) or H-RasV12 effector domain mutants (35S or 40C) were resolved by 10% SDS-PAGE, transferred to PVDF membranes and probed with phospho-specific antibodies against pY705 STAT3 (pY STAT3), pS727 STAT3 (pS STAT3), pY204 ERK (pERK), pS473 Akt (pAKT), or total STAT3, ERK, AKT and tubulin, as indicated. Protein lysate from mouse liver 4 h following an injection with 30µg of lipopolysaccharide was used as a positive control (+ve). (**B**) H-RasV12 transformed MEFs were treated with DMSO (unt), Rapamycin (10nM), LY294002 (50µM) or PD0325901 (1µM) for 2 h. Cell lysates were processed for immunoblotting and probed with phospho-specific antibodies against pS727 STAT3 (pS STAT3), pT389 p70 S6 Kinase, pY204 ERK (pERK), pS473 Akt (pAKT), or total STAT3 and tubulin as loading controls. (**C**) H-RasV12 transformed MEFs were treated with the indicated dose of PD0325901 or vehicle (DMSO) for 2 h. Cell lysates were processed for immunoblotting and probed with phospho-specific antibodies against pS727 STAT3 (pS STAT3), pY204 ERK (pERK), pS473 Akt (pAKT), or total STAT3 and total ERK as loading controls. (**D**) N-Ras transformed human THP-1 acute myelocytic leukemia cells were treated with the indicated doses of PD0325901 (nM) or vehicle (DMSO) for 2 h prior to the preparation of cell lysates for immunoblotting. Lysates were probed for the indicated proteins and phospho-proteins as described in panel (C).

To address this question, we employed H-Ras mutants that selectively activate individual Ras effector pathways [21,22]. Activating mutations in Ras (at codons 12, 13 or 61) lock it in a GTP bound (active) state, while a second mutation in the effector domain restricts Ras signaling to a single downstream pathway (i.e., the 35S mutation restricts H-Ras signaling to the Raf-MEK-ERK pathway; and the 40C mutation signals through the PI3K pathway) [23,24]. We generated stable cell lines expressing mutant H-RasV12, H-RasV12/35S or H-RasV12/40C and confirmed that the H-Ras mutations were activating the expected downstream kinase pathways. H-RasV12 activated both the Raf-MEK-ERK pathway (as shown by the phosphorylation of ERK) and the PI3K pathway (as shown by the phosphorylation of Akt). H-RasV12/35S preferentially signaled through Raf-MEK-ERK, inducing ERK phosphorylation (Figure 1A, lane 3), whereas H-RasV12/40C activated the PI3K-Akt pathway, inducing AKT phosphorylation but not ERK phosphorylation (Figure 1A, lane 4).

Consistent with our previous data we found that activation of Ras (H-RasV12) led to S727 phosphorylation, but not Y705 phosphorylation of STAT3 (Figure 1A). Interestingly, we detected STAT3 S727 phosphorylation in cells where only the Raf-MEK-ERK pathway was active, but not in cells with activated PI3K alone, in spite of clear evidence of activation of AKT (Figure 1A).

In addition to phosphorylation at S727 of STAT3, these Ras-transformed cells expressed phosphorylated forms of ERK, S6K, and AKT (Figure 1A, B). To examine the role of kinase signaling pathways in STAT3 phosphorylation, we treated cells with selective kinase inhibitors. Inhibition of the MEK-ERK pathway by using the MEK inhibitor PD0325901 impaired S727 phosphorylation, along with ERK phosphorylation, as expected (Figure 1B, lane 4). However, pS727 was unaffected by inhibition of mTOR with rapamycin or inhibition of the PI3K-AKT pathway with LY294002, in spite of effective inhibition of their characteristic targets, S6K and AKT (Figure 1B, lanes 2 and 3). 

To confirm that the Raf-MEK-ERK pathway is engaged by activated Ras to transduce phosphorylation of STAT3 on S727, we titrated the dose of the MEK-ERK pathway inhibitor, PD0325901 [25]. Treatment of H-RasV12 transformed MEFs with increasing concentrations of PD0325901 from 50-1000 nM impaired ERK phosphorylation, but had no impact on AKT phosphorylation (Figure 1C). Importantly, treatment with doses of PD0325901 ranging from 50-1000nM completely inhibited S727 phosphorylation of STAT3, indicating that the Raf-MEK-ERK pathway is necessary for STAT3 phosphorylation in the context of Ras-transformation.

**Figure 2:**
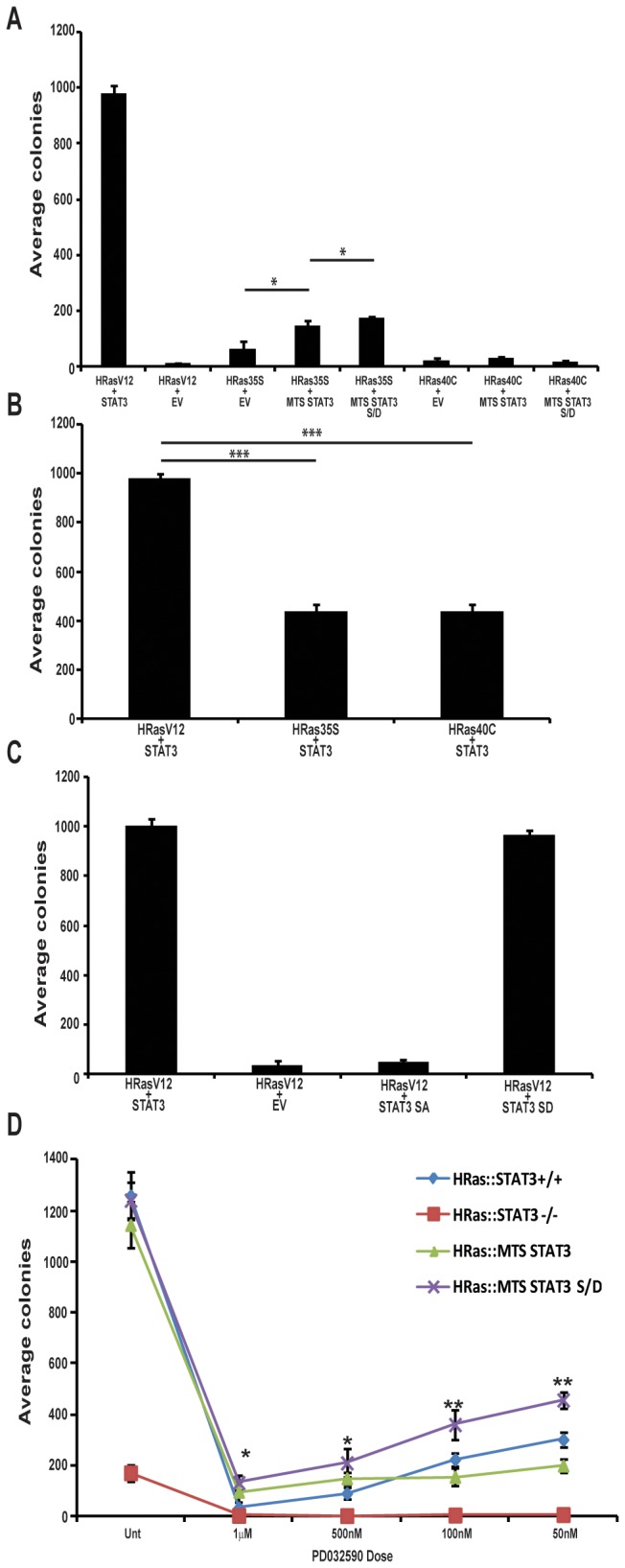
H-RasV12 transformation requires the MEK-ERK pathway and S727 phosphorylation of mitochondrial STAT3. (**A**) STAT3^-/-^ MEFs were stably transduced with H-RasV12 and empty vector (EV) or wild-type STAT3 (WT STAT3). STAT3^-/-^ MEFs were also transduced with H-RasV12 effector domain mutants (35S to activate the MEK-ERK pathway and 40C to activate the PI3K pathway) in addition to a mitochondrially restricted STAT3 (35S or 40C+MTS STAT3) or a mitochondrially restricted phospho-S727 mimetic STAT3 (35S or 40C+MTS STAT3 S/D). 10^3^ cells were plated in media supplemented with 0.3% agar and colonies counted 14 d later. (**B**) Wild type MEFs were stably transduced with H-RasV12, H-Ras35S or H-Ras40C and 10^3^ cells were plated in media supplemented with 0.3% agar and colonies counted 14 d later. (C) STAT3^-/-^ MEFs were stably transduced with H-RasV12 and wild-type STAT3 (H-RasV12+STAT3) empty vector (H-RasV12+EV), S727A STAT3 (H-RasV12+SA) or S727D STAT3 (H-RasV12+SD). 10^3^ cells were plated in media supplemented with 0.3% agar and colonies counted 14 d later. (**D**) H-RasV12 transformed wild-type (H-Ras:STAT3+/+), STAT3-/- MEFs (H-Ras:STAT3-/-) or H-Ras:STAT3-/- MEFs stably transduced with mitochondrially restricted STAT3 (H-Ras:MTS-STAT3) or mitochondrially restricted phospho-S727 mimetic mutant STAT3 (H-Ras:MTS-STAT3 S/D) cells were plated in media supplemented with 0.3% agar and the indicated dose of PD0325901 or vehicle (unt) and colonies counted 14 d later. Each point represents the mean of biological triplicates, error bars are ±1 standard deviation from the mean. Student’s t-test was used to determine significant differences. * denotes p<0.05, ** denotes p<0.01, *** denotes p<0.0001.

Phosphorylation of STAT3 on S727 in Ras-transformed MEFs and its inhibition by treatment with MEK inhibitors provided strong evidence for a role for Ras-activated MAPK in STAT3 S727 phosphorylation. To extend these results from experimental murine cells to human tumor cells, we examined the STAT3 S727 phosphorylation and its inhibition by PD0325901 in the human acute myelocytic leukemia cell line, THP-1, which express mutant N-Ras [26]. Extracts from THP-1 cells displayed constitutively phosphorylated ERK (Figure 1D, panel 3, lane 1), as expected for N-Ras-transformed cells. In addition, these cells expressed STAT3 constitutively phosphorylated on S727 (Figure 1D, panel 1, lane 1). Treatment of cells with concentrations of the MEK inhibitor PD0325901 between 50 and 1000 nM led to the inhibition of phospho-ERK (Figure 1D, panel 3, lanes 2-5) and the simultaneous loss of STAT3 pS727 (panel 1, lanes 2-5). Similar to the effects of MEK inhibition observed in mouse cells, treatment of THP-1 cells with PD0325901 strongly inhibited pERK at 50 nM and completely inhibited its phosphorylation at higher doses, without affecting total ERK protein levels. STAT3 pS727 was completely inhibited by all tested doses of PD0325901 without affecting total STAT3 expression, again paralleling the observations in mouse cells (compare Figure 2C and 2D). These data support the conclusion that Ras-dependent STAT3 S727 phosphorylation is MEK dependent in tumors of both mouse and human origin.

### The Raf-MEK-ERK but not the PI3K-Akt pathway co-operates with mitochondrial-STAT3 in Ras-mediated transformation

We previously found that Ras transformation is dependent on a mitochondrial pool of STAT3, and that S727 phosphorylation of this pool of STAT3 is critical for maintaining a transformed phenotype [12]. Therefore, to define whether Ras activation of the Raf-MEK-ERK pathway and the consequent S727 phospohorylation of the mitochondrial pool of STAT3 was important for transformation, we performed colony assays as a measure of anchorage independent cell growth. STAT3-null cells stably expressing the H-Rasv12 effector domain mutations were reconstituted with either wild-type STAT3, a STAT3 mutant that is exclusively expressed in mitochondria, or mitochondrial STAT3 harboring a serine to aspartic acid point mutation intended to mimic phosphorylation of S727. These cells were plated in soft agar to measure anchorage independent growth. Consistent with our previous data, we observed that H-RasV12 transformed MEFs formed colonies in soft agar, while loss of STAT3 largely abolished anchorage independent growth (Figure 2A). We next addressed whether H-RasV12 was capable of maintaining a transformed phenotype when restricted to signaling individually through the Raf-MEK-ERK or the PI3K pathway. Therefore, we plated MEFs stably expressing H-RasV12/35S or H-RasV12/40C in soft agar. Similar to H-RasV12, neither H-RasV12/40C nor H-RasV12/35S supported colony formation in the absence of STAT3 (Figure 2A, compare bar 1 to 3 and 6). However, when H-RasV12/35S-expressing STAT3^-/-^ MEFs were engineered to express a mutant form of STAT3 exclusively localized to mitochondria (MTS-STAT3), they formed significantly (p<0.05) more colonies than RasV12/35S transduced, STAT3^-/-^ MEFs (Figure 2A, compare bar 3 to 4). In contrast, the mitochondrial form of STAT3 did not increase colony formation in H-RasV12/40C transduced cells (Figure 2A, compare bars 6, 7 and 8).

We have previously shown that STAT3 S727 phosphorylation supports its mitochondrial activity, which is necessary for Ras transformation. To address whether constitutive phosphorylation at this residue could increase transformation, we generated a mitochondrially-targeted STAT3 that harbored a phospho-mimetic aspartic acid substitution of S727 (MTS-STAT3S/D). Introducing this mutation into STAT3^-/-^ MEFs expressing H-RasV12/35S enabled growth in soft agar (Figure 2A, compare bar 3 to 5). Indeed, these cells formed modest but statistically significant (p<0.05) more colonies than H-RasV12/35S+MTS-STAT3 cells (compare bars 4 and 5). However, H-RasV12/40C expressing cells did not support colony growth no matter which STAT3 construct was introduced. The inability of H-RasV12/40C to support anchorage independent growth suggests that activation of the PI3K pathway is not sufficient to transform these cells in the presence or absence of mitochondrial STAT3. In addition, it would appear that the presence of S727 STAT3 phosphorylation, at least to the extent that this feature can be mimicked by aspartate substitution, is insufficient to augment transformation by PI3K pathway activation. We note that this data also indicate that over-expression of MTS-STAT3 S727D alone is not transforming, even in the presence of activated PI3K signaling. Together these data indicate that activation of the MEK-ERK pathway is central to the dependence on STAT3 for cell transformation by Ras and that phosphorylation of mitochondrial STAT3 is an important target. However, while STAT3 S727 phosphorylation contributes to cell transformation, activation of mitochondrial STAT3 is not sufficient to replace the major contributions of the activated ERK cascade to anchorage independent growth.

### Both mitochondrial and non-mitochondrial STAT3 pools contribute to Ras transformation

The ability of mitochondrially-restricted MTS-STAT3 and MTS-STAT3-S/D to support modest anchorage independent growth of H-RasV12/35S-expressing cells prompted us to examine the role of total STAT3. STAT3-null cells were reconstituted with STAT3 and transformed with H-RasV12 and the S35 or 40C effector domain mutants. As observed previously [12], both effector domain-mutant constructs were capable of diminished transformation in the presence of STAT3 (Figure 2B). Interestingly, the 40C mutant activating PI3K signaling was significantly transforming in the presence of wild type STAT3, compared to its inability to transform cells expressing only mitochondrial STAT3 (compare Figure 2A and 2B). In contrast, the 35S mutant activating MEK-ERK signaling was at best 2-fold more transforming in the presence of a total cellular pool of STAT3 versus a mitochondria-only pool.

To probe the role of S727 phosphorylation in Ras transformation, we also assessed anchorage independent growth of cells expressing STAT3-S727A phospho-mutant and STAT3-S727D phospho-mimetic constructs. H-RasV12 was unable to transform cells lacking STAT3 or expressing STAT3-S727A (Figure 2C). In contrast, STAT3-null cells reconstituted with STAT3-S727D phospho-mimetic were transformed to an extent equal to wild type STAT3. STAT3-S727D was not transforming on its own, in the absence of H-RasV12 (data not shown).

Taken together, the results shown in Figure 2A-C suggest that the activated MEK-ERK pathway in Ras transformed cells is substantially dependent on mitochondrial STAT3 in its S727 phosphorylated state. However, transformation by the activated PI3K pathway, while dependent on the expression of STAT3, is not mediated by its mitochondrial form. This result is reminiscent of our previous observation that activation of the Ral pathway is also dependent on a non-mitochondrial but probably non-nuclear function of STAT3 [12]. 

### The Raf-MEK-ERK pathway is necessary for S727 phosphorylation of STAT3 and transformation

The data described above document that H-Ras activation of MEK-ERK signaling results in phosphorylation of STAT3 on S727, and that the expression of a mitochondrially-restricted phospho-mimetic STAT3 supports Ras transformation. However, these data do not address directly the requirement of mitochondrial S727 phosphorylation in Ras-transformation. If STAT3 S727 phosphorylation is a critical MEK-ERK target, then inhibition of this pathway in H-RasV12 transformed cells should impede colony formation. Moreover, if this phosphorylation event is a major ERK pathway target, then MTS-STAT3S727D mutation might be expected to support cell transformation even in the presence of MEK-ERK inhibition. To test this hypothesis, we performed colony assays on a panel of H-RasV12 transformed cells expressing wild-type STAT3, no STAT3, MTS-STAT3 or MTS-STAT3 S/D in the presence of increasing titrating concentrations of the MEK inhibitor, PD0325901.

PD0325901 treatment was sufficient to fully block ERK phosphorylation at the higher doses tested (0.5-1µM) and to substantially impair ERK phosphorylation at the lowest dose (50nM) (Figure 1C). Vehicle treated H-RasV12:STAT3^+/+^, H-RasV12:MTS-STAT3 and H-RasV12:MTS-STAT3S/D cells formed equivalent colony numbers when cultured in soft agar, while H-RasV12 cells lacking STAT3 formed almost no colonies (Figure 2D). The highest PD0325901 dose (1µM) resulted in a dramatic decrease in colony formation in all cell lines, which confirmed that the Raf-MEK-ERK pathway is critical for transformation by H-RasV12. However, a modest but statistically significant increase in colonies formed with cell lines expressing phospho-mimetic mitochondrial STAT3 (H-RasV12:MTS-STAT3S/D) compared to wild-type STAT3 through much of the MEK inhibition titration, even at the highest dose of PD0325901. Whilst the H-RasV12: STAT3^+/+^ and H-RasV12:MTS-STAT3 were similarly sensitive to inhibitor throughout the dose range, the difference between these cell lines and the H-RasV12:MTS-STAT3S/D line became increasingly apparent as the dose of PD0325901 was titrated down from 1000 to 50nM (p<0.01) (Figure 2D). Taken together, these data confirm that the MEK-ERK pathway is essential for H-RasV12 transformation and that this requirement is partly due to targeted phosphorylation of S727 of the mitochondrial STAT3 pool.

### The Raf-MEK-ERK signaling is necessary for S727 phosphorylation of STAT3 and transformation by K- and N-Ras

Activating mutations in H-Ras occur in bladder, testicular and thyroid cancers, but are rare in other human cancers. The overall prevalence of H-Ras mutation in cancer is small compared to K- and N-Ras mutations [27]. Mutant K- and N-Ras are observed in approximately 20% of all human cancers, making them amongst the most prevalent activated proto-oncogenes. Despite having 85% sequence identity, the distinct Ras oncoproteins differ in both their level of expression and in a critical C-terminal hyper-variable region, which is subject to alternate farnesyl or palmitoyl lipidation controlling subcellular localization and signaling strength [28]. We previously showed that N-, K- and H-Ras all require the mitochondrial activity of STAT3 for transformation. Because N-, K- and H-Ras can all engage the Raf-MEK-ERK pathway, it was of interest to test whether pathway activation was critical for mitochondrial STAT3 S727 phosphorylation and transformation.

**Figure 3:**
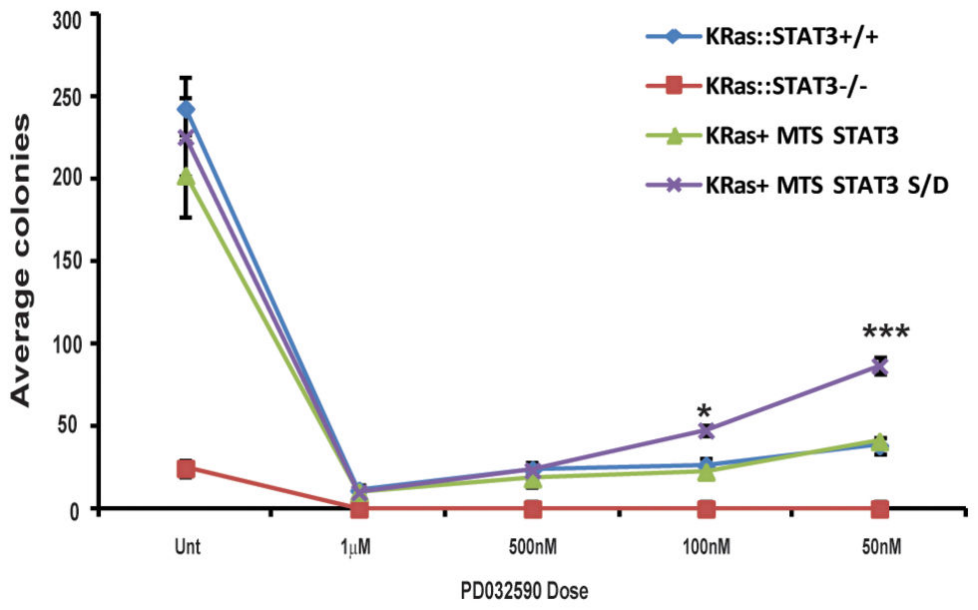
K-RasG12V transformation requires the MEK-ERK pathway and S727 phosphorylation of mitochondrial STAT3. K-RasV12 transformed wild-type (KRas), STAT3-/- MEFs (KRas:STAT3-/-) or KRas:STAT3-/- MEFs stably transduced with mitochondrially restricted STAT3 (KRas:STAT3-/-+MTS-STAT3) or mitochondrially restricted phospho-S727 mimetic mutant STAT3 (KRas: STAT3-/- +MTS-STAT3 S/D) cells were plated in media supplemented with 0.3% agar and the indicated dose of PD0325901 or vehicle (unt) and colonies counted 14 d later. Each point represents the mean of biological triplicates, error bars are ±1 standard deviation from the mean. We used Student’s t-test to determine significant differences. * denotes p<0.05, *** denotes p<0.001.

**Figure 4:**
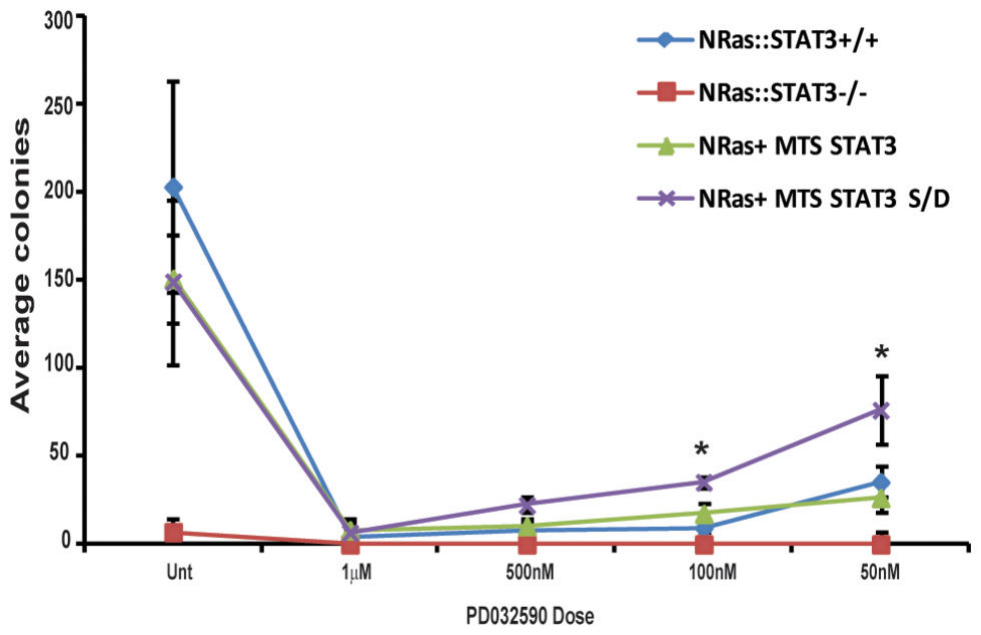
N-RasG12V transformation requires the MEK-ERK pathway and S727 phosphorylation of mitochondrial STAT3. N-RasV12 transformed wild-type (NRas), STAT3-/- MEFs (NRas:STAT3-/-) or NRas:STAT3-/- MEFs stably transduced with mitochondrially restricted STAT3 (NRas: STAT3-/- + MTS-STAT3) or mitochondrially restricted phospho-S727 mimetic mutant STAT3 (NRas: STAT3-/- + MTS-STAT3 S/D) cells were plated in media supplemented with 0.3% agar and the indicated dose of PD0325901 or vehicle (unt) and colonies counted 14 d later. Each point represents the mean of biological triplicates, error bars are ±1 standard deviation from the mean. We used Student’s t-test to determine significant differences.* denotes p<0.05.

To address this issue, we generated wild type or STAT3^-/-^ MEFs stably expressing either N-RasG12V or K-RasG12V oncogenes. Ras expressing STAT3^-/-^ cells were complemented with mitochondrially-targeted STAT3 or the mitochondrially-targeted S727D substitution mutant described earlier. These cells were plated in soft agar supplemented with either vehicle or with titrating doses of PD0325901. Consistent with data observed with H-Ras, transformation by K- (Figure 3) or N-Ras (Figure 4) required STAT3, and inhibition of the MEK-ERK pathway with PD0325901 significantly reduced anchorage independent growth.

K- or N-Ras transformed MEFs expressing wild type STAT3 or mitochondrially-targeted STAT3 behaved indistinguishably from each other in their clonogenic capacity in response to MEK inhibition. However, there was a statistically significant (p<0.001 for K-Ras and p<0.05 for N-Ras) increase in colony forming capacity of K- or N-Ras transformed MEFs expressing mitochondrially-targeted S727D mutant STAT3 in the presence of 50 and 100 nM PD0325901 (Figure 3 and Figure 4). While anchorage independent growth was still impaired in the presence of phospho-mimetic mitochondrial STAT3 at high concentrations of the MEK inhibitor, colony formation was inhibited only approximately 2-fold at 50 nM PD0325901, a concentration that abrogated anchorage independent growth of wild type STAT3-expressing cells. Together, these data show that serine 727 phosphorylated STAT3 is necessary for transformation by all 3 Ras isoforms, H, K and N. These data also show that the Raf-MEK-ERK pathway, and not the PI3K pathway triggered by Ras oncoproteins leads to STAT3 S727 phosphorylation. Moreover, these data suggest that maintenance of mitochondrial STAT3 phosphorylation represents an important component of Ras transformation and provision of this modification partially relieves the dependence on activated ERK pathway for anchorage independent growth.

## Discussion

STAT3 is over-expressed and/or post-translationally modified in the majority of human cancers. The current dogma for the contribution of STAT3 to cancer biology is as a potent transcription factor driving expression of genes controlling numerous hallmark features of tumor cells [2]. This paradigm is clearly implicated for oncogenes that target Y705 phosphorylation of STAT3 (e.g., JAK2, EGFR, NPM-ALK), but it is less clear why STAT3 would be important for non-tyrosine kinase oncogenes that do not lead to STAT3 tyrosine phosphorylation (e.g., H-, N-, K-Ras and c-Myc). Nonetheless, we recently showed that the Ras family of oncogenes requires STAT3 expression to transform cells. Importantly, this requirement for STAT3 was found to be due to a mitochondrial pool of STAT3 that augments the activity of the electron transport chain, lactate dehydrogenase activity, and ATP abundance [12]. The mitochondrial activity of STAT3 and Ras-mediated transformation is dependent on the phosphorylation of STAT3 on S727. Many kinases have been shown to phosphorylate STAT3 on S727 in a stimulus dependent manner (e.g., ERK, JNK, p38, mTOR, SEC1, PKCδ, and Rac- and VAV-dependent kinases [8,29]). However, it has not been documented which serine kinase(s) activated by the Ras oncogenes are responsible for serine phosphorylation of STAT3. In this study, we provide experimental evidence that the ERK pathway is responsible and critical for this modification.

### Isolated Ras-activated pathways cannot transform STAT3 null cells

Ras engages multiple cell signaling pathways to elicit cellular responses, and oncogenic Ras exploits these pathways for malignant transformation. The best characterized in the context of cancer are the Raf1-MEK-ERK, PI3K, and Ral pathways. Deregulated PI3K signaling (e.g., loss of the negative regulator PTEN) is one of the most commonly mutated tumor suppressors seen in cancer, particularly malignancies of the central nervous system and endometrium [30]; constitutive Raf signaling (e.g., Raf600E mutation) drives approximately 60% of melanoma [31]; elevated Ral expression is seen in colon, bladder and prostate cancers [32]; and RalGDS activation of Ral has been implicated in Ras-driven skin cancer [33]. 

Previous data have shown that strong constitutive activation of each of these pathways in isolation can transform MEFs, but not as potently as activated H-Ras. However, like the Ras oncogenes themselves, activation of each individual pathway could not transform STAT3^-/-^ MEFs [12]. As confirmed and expanded in this study, activation of the Raf-MEK-ERK pathway in the absence of STAT3 led to significantly fewer soft agar colonies compared to cells engineered to express mitochondrially restricted STAT3 (Figure 2A). Moreover, cells expressing a mitochondrially restricted STAT3 mutated to mimic phosphorylation at S727 (S727D) formed modestly but statistically significantly more colonies than those expressing wild type mitochondrial STAT3. In contrast, limited cell transformation observed in the presence of activated PI3K was not increased by mitochondrial STAT3, whether or not S727 phosphorylation was mimicked. These data demonstrate that the mitochondrial pool of STAT3 can support transformation in cells with activated ERK, but not PI3K.

### Ras induced phosphorylation of STAT3 S727 through an ERK dependent pathway supports transformation

Whilst STAT3 S727 can be phosphorylated by many kinases, ERK is the most common kinase implicated in this modification [8]. ERK2 and STAT3 interact [13] and the use of MEK-ERK inhibitors blocks STAT3 S727 phosphorylation in response to IL-2 [14]. In this study, we showed that STAT3 is phosphorylated on S727 but not on Y705 in the presence of activated H-Ras. Moreover, we showed that inhibition of the MEK-ERK pathway with PD0325901 reduced STAT3 S727 phosphorylation to undetectable levels. Inhibition of STAT3 S727 phosphorylation was observed at the lowest concentrations of PD0325901 tested (50nM), at which ERK phosphorylation was largely though not completely inhibited. This dose-response relationship suggests that effective ERK activation is required for STAT3 phosphorylation.

The requirement of the ERK pathway for H-Ras transformation was also observed for K- and N-Ras transformation, as indicated by the sensitivity of anchorage independent cell growth to PD0325901. Inhibition of the MEK-ERK pathway blocked colony formation driven by all three activated Ras isoforms (Figures 2D, 3 and 4). To directly test the contribution of S727 phosphorylated mitochondrial STAT3, we generated cells that exclusively expressed a mitochondrially restricted STAT3 harboring a serine to aspartic acid mutation to mimic phosphorylation at S727. These cells were sensitive to H-, K-, and N-Ras-mediated transformation. Moreover, they were significantly less sensitive to MEK inhibition when compared to cells expressing wild type STAT3, when tested for anchorage independent cell growth. However, in the absence of MEK inhibition, MTS-STAT3-S/D and total STAT3-S/D expressing cells were equivalently responsive to Ras transformation as cells expressing wild type STAT3. These data suggest that activated Ras is sufficient to maximally phosphorylate mitochondrial STAT3, at least to an extent that saturates any function required for transformation. It is interesting to note that the MTS-STAT3-S/D mutant did not lead to an increase in the colony formation of Ras transformed cells in the absence of PD0325901. It was also not transforming on its own. We hypothesize that phospho-S727 STAT3 contributes to transformation by synergizing with additional MEK-dependent targets required for transformation, probably by supporting a metabolic shift necessary for the transformed state.

MTS-STAT3-S727D cells expressing H-, K- and N-Ras were less sensitive to inhibition of anchorage independent growth following PD0325901 treatment; however they were only partially resistant to kinase inhibition. We observed that the lowest concentration of PD0325901 tested resulted in about a 2-fold reduction in the number of colonies in MTS-STAT3-S/D cells compared to vehicle treated cells. This PD0325901 concentration was not sufficient to completely abolish ERK phosphorylation, although it did reduce phosphorylation of wild type STAT3-S727 to undetectable levels. We therefore conclude that the additional reduction of clonogenicity was due to inhibition of other targets of ERK. Indeed, ERK has more than 200 documented targets [34], so it is not surprising that STAT3 is not the only important target necessary for transformation. In addition, expression of MTS-STAT3-S/D did not increase the ability of activated PI3K signaling to transform cells, although total STAT3 stimulated PI3K-dependent transformation. This observation suggests that while mitochondrial STAT3 is a necessary ERK phosphorylation target, other functions of STAT3 in conjunction with additional PI3K mediators also contribute to Ras transformation.

Mitochondrial functions of STAT3 include STAT3 interaction with and augmentation of the activity of the electron transport chain complexes I, II and V [12,15]. STAT3 also suppresses the opening of the mitochondrial transition pore in cardiac tissue [35]. All these activities are dependent on S727 phosphorylation of STAT3. The Warburg effect, a phenomenon in which transformed cells favor aerobic glycolysis over oxidative phosphorylation for the production of ATP [17], also appears to depend on mitochondrial STAT3, at least in Ras transformed cells [12,15]. It has become clear that mitochondria play essential roles in transformation, which are likely to be more dependent on anaplerosis than on ATP generation [36]. We anticipate that activated mitochondrial STAT3 facilitates anaplerosis, and hence provides a survival advantage to transformed cells, but is not sufficient to transform on its own. Therefore, it is not surprising that the STAT3 S727D mutation was not able to transform cells alone. Nonetheless, the sensitivity of Ras-transformed cells to growth inhibition by the MEK inhibitor PD0325901 and its partial reversal by mimicking STAT3 S727 phosphorylation, demonstrates the importance of STAT3 as one critical MEK-ERK target during transformation and suggests that inhibition of mitochondrial STAT3 activity could be a promising therapeutic approach to Ras-dependent cancers.
