# Correction: The MEK-ERK Pathway Is Necessary for Serine Phosphorylation of Mitochondrial STAT3 and Ras-Mediated Transformation

**DOI:** 10.1371/annotation/5b4e222a-a9bc-4036-882e-cd975301ca89

**Published:** 2013-12-17

**Authors:** Daniel J. Gough, Lisa Koetz, David E. Levy

Some information was missing from the funding section. The correct funding statement should read: This study was supported in part by grant AI28900 from the National Institutes of Health, grant 3401-11 from the Leukemia and Lymphoma Society and grant 1024929 from the National Health and Medical Research Council. The funders had no role in study design, data collection and analysis, decision to publish, or preparation of the manuscript.

Both Daniel Gough and David Levy are corresponding authors. Dr. Gough can be reached here : daniel.gough@monash.edu

